# Acute urinary retention during menstruation: Questions

**DOI:** 10.1007/s00467-020-04637-w

**Published:** 2020-06-18

**Authors:** Hannah Jeffery, Swati Jha

**Affiliations:** 1 Department of Paediatric Surgery, Sheffield Children’s Hospitals, Sheffield, UK; 2 Department of Urogynaecology, Sheffield Teaching Hospitals, Level 4 Jessop Wing, Tree Root Walk, Sheffield, S10 2SF UK

## Case report

**Fig. 1:**
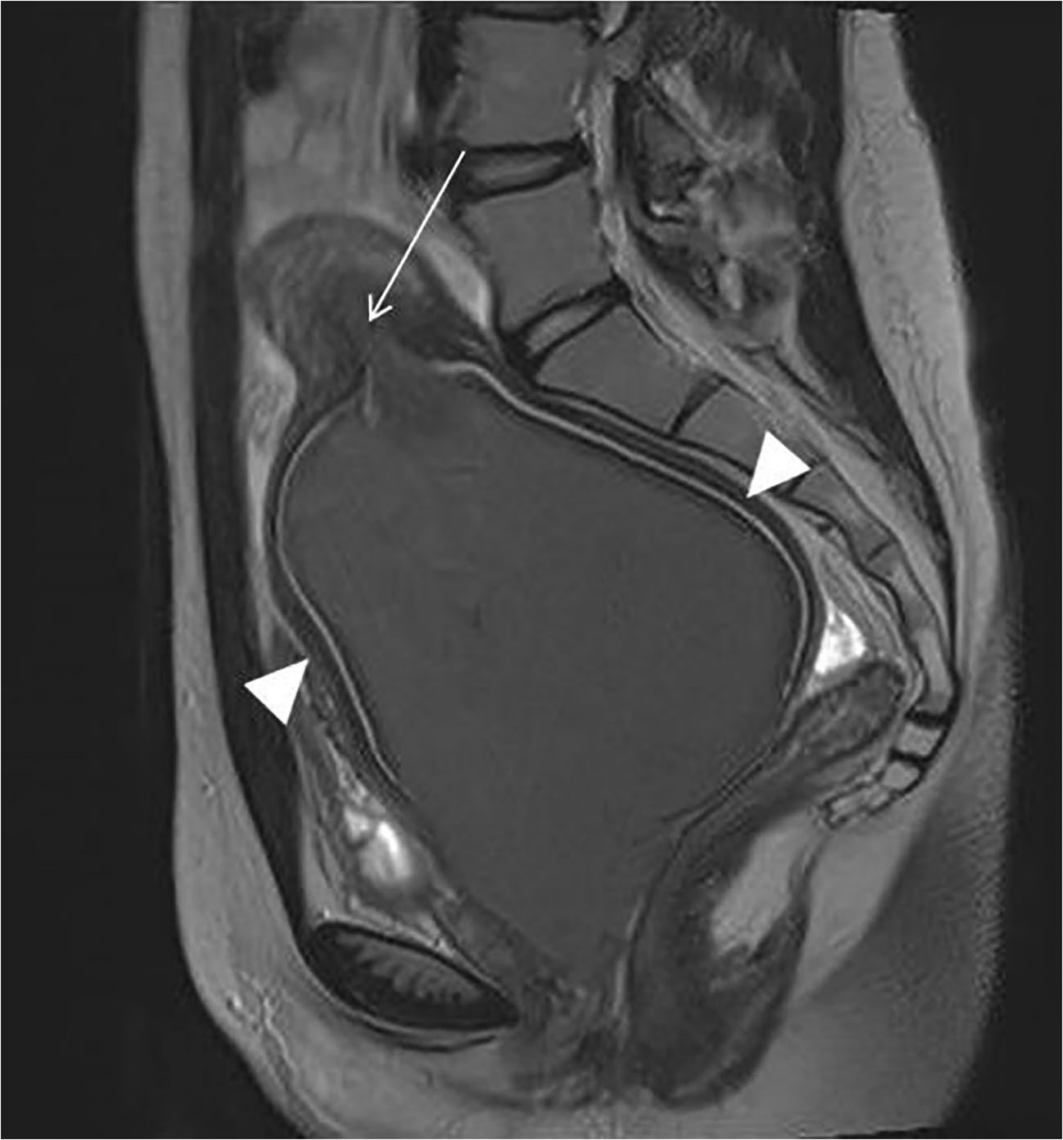
T2-weighted sagittal image of the distended cervix. The uterine cavity (arrow) is only slightly distended. The cervical stroma (arrow heads) is considerably stretched

A 15-year-old girl presented to the emergency department with acute urinary retention during menstruation. She complained of severe dysmenorrhoea from menarche at age 12 and reported difficulty emptying her bladder during menstruation over the 6 months prior to her acute presentation. On admission, an indwelling catheter was inserted and over 1.5 l of urine drained. Renal functions were within normal ranges. Initially, an ultrasound scan was performed but was difficult to interpret so an MRI was undertaken. The MRI showed the absence of the right kidney and a uterine didelphys. There was a massive right sided haematocolpos (measuring 10.5 cm) and haematometra secondary to an obstructed hemi vagina. Figure 1 shows the grossly dilated cervix and loss of normal cervical anatomy, with the obstructed hemivagina and the cervical canal forming a continuous tract. On the left, the patent vagina was laterally displaced by the obstructed hemi vagina; the uterus appeared normal.

She was referred to the local teaching hospital where the diagnosis was made and surgery undertaken to correct the problem. She had an overnight hospital stay following surgery and periods as well as voiding returned to normal after correction of the obstruction. A follow-up scan showed a uterine didelphys with both uteri a normal size and no other vaginal abnormality.

## Questions

1. What is the diagnosis and how does this usually present?
2. How should this condition be managed?
3. What are the potential complications?

